# Successful treatment of β-catenin-positive, immune checkpoint inhibitor-resistant tumor in a case of multicentric hepatocellular carcinoma

**DOI:** 10.1007/s12328-025-02266-0

**Published:** 2026-03-21

**Authors:** Hirotaka Nomiya, Hidetaka Matsuda, Takuto Nosaka, Yu Akazawa, Tomoko Tanaka, Kazuto Takahashi, Tatsushi Naito, Masahiro Ohtani, Yoshiaki Imamura, Yasunari Nakamoto

**Affiliations:** 1 Second Department of Internal Medicine, Faculty of Medical Sciences, University of Fukui, 23-3 Matsuoka Shimoaizuki, Eiheiji-Cho, Yoshida-Gun, Fukui 910-1193 Japan; 2 Department of Gastroenterology, Fukui General Hospital, 28-16-1, Egami-Cho, Fukui-City, Fukui Japan; 3 Department of Pathology, University of Fukui Hospital, 23-3 Matsuoka Shimoaizuki, Eiheiji-Cho, Yoshida-Gun, Fukui Japan

**Keywords:** Hepatocellular carcinoma, Multicentric occurrence, Immune checkpoint inhibitor, β-catenin, Proton beam therapy

## Abstract

Multicentric hepatocellular carcinoma (HCC) often exhibits a heterogeneous inter-nodal response to systemic therapy, leading to reduced therapeutic effectiveness. Here, we report the case of a 70-year-old woman with multicentric HCC treated with atezolizumab plus bevacizumab, followed by lenvatinib. While most nodules responded well to systemic therapy, one nodule at S7 exhibited progression. Distinct from the histology of another nodule that responded to atezolizumab plus bevacizumab, percutaneous ultrasound-guided biopsy of this progressive nodule revealed nuclear positivity for β-catenin. This finding suggested a potential role of β-catenin in immune checkpoint inhibitor resistance. Proton beam therapy was subsequently administered to the resistant lesion, achieving local control. The continuation of immune checkpoint therapy effectively maintained remission of the other nodules, and the patient remained in long-term remission without recurrence. This case indicates the importance of molecular profiling, including β-catenin expression, in cases of multicentric HCC to predict immune checkpoint inhibitor resistance and guide treatment strategies for each nodule. Additionally, it demonstrates the potential of proton beam therapy as a targeted therapeutic approach for immune checkpoint inhibitor-resistant nodules. These findings highlight the possible need for personalized treatment in multicentric HCC by integrating molecular and immunological insights to predict outcomes for complex tumor biology.

## Introduction

Primary liver cancer ranks sixth in incidence and third in cancer-related mortality worldwide [1]. Approximately 80% of liver malignancies are hepatocellular carcinoma (HCC), which is characterized by aggressive behavior and poor prognosis [2, 3]. Multicentric occurrence (MO) is one reason for poor prognosis [4]. Notably, 50–75% cases of HCC are multifocal from the time of diagnosis [5]. Therefore, establishing effective treatment strategies for MO-HCC is crucial for improving the long-term prognosis of these patients.

In recent years, combination of the anti–programmed death ligand 1 (PD-L1) antibody, atezolizumab, with the endothelial growth factor (VEGF) inhibitor, bevacizumab, has emerged as the standard of care for unresectable HCC [4, 6, 7]. This combination therapy has demonstrated remarkable efficacy; however, some patients with HCC fail to benefit from this treatment strategy, highlighting the need to identify factors influencing treatment response. Understanding the mechanisms of resistance has therefore become crucial, and a significant emphasis has been placed on the exploration of biomarkers to predict treatment efficacy. Notably, the Wnt/β-catenin signaling pathway has emerged as a potentially valuable indicator for predicting immunological resistance to anti-PD-1 therapy in HCC [8–10].

Moreover, there is a need for the development of a subsequent therapeutic approach to address the issue of immune checkpoint inhibitors (ICI) resistance in HCC [11]. Herein, we describe a patient with MO-HCC who exhibited a heterogeneous inter-nodal response to ICIs. In this case, a correlation between tumoral β-catenin expression and ICI resistance was suggested. Ultimately, additional proton beam therapy (PBT) proved effective for treating the remaining tumor.

## Case report

A 70-year-old woman presented to our hospital for a thorough examination of liver nodules. She had a documented medical history of alcoholic liver disease diagnosed at the age of 60 years. Her alcohol intake history included consumption of 40 g of pure ethanol daily for 50 years. She had an allergy to iodine-based contrast agents. In X-4 years, she underwent abdominal computed tomography (CT) scan, during which micronodules were detected in bilateral lobes of the liver. In addition, two small nodules (< 10 mm) showing strong enhancement in the arterial phase were detected in S4, S6 on gadolinium-ethoxybenzyl-diethylenetriamine pentaacetic acid enhanced magnetic resonance imaging (Gd-EOB MRI). Since then, she had undergone periodic imaging examinations. In year X, she was admitted to our hospital for close examination and treatment after an abdominal CT scan revealed that several nodules had grown over 4 years.

**Fig. 1:**
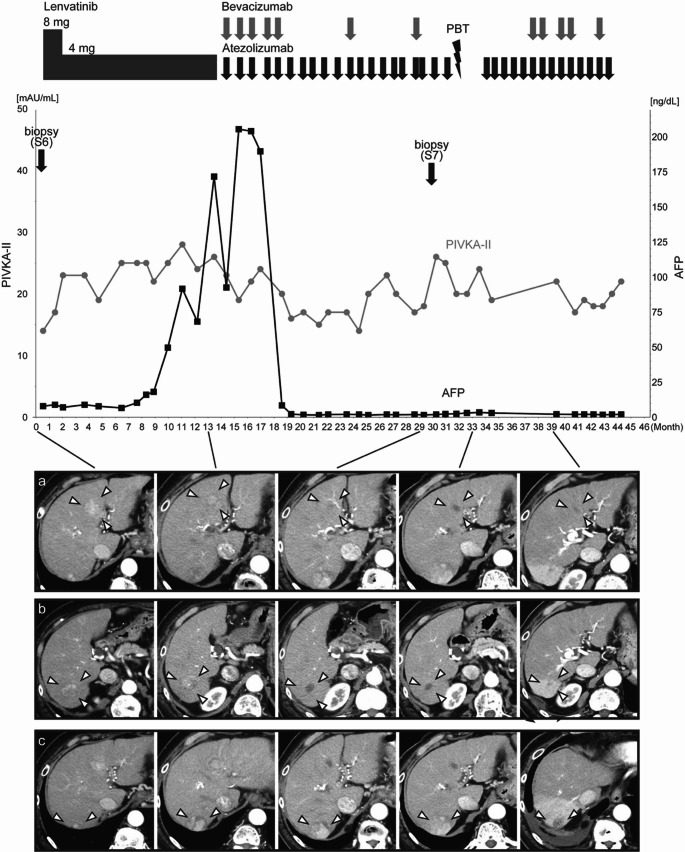
Clinical course of the patient. This figure demonstrates the temporal changes in the target nodules observed on contrast-enhanced computed tomography (CT) scans (arterial phase) and tumor marker levels in response to various treatments. The upper, middle, and lower panels (a, b, and c) display the S4, S6, and S7 nodules, respectively. These panels demonstrate the temporal changes in the different hepatocellular carcinoma (HCC) nodules in response to various treatments. **a** The S4 and **b** S6 nodules showed reduced contrast enhancement following the initiation of lenvatinib treatment. The subsequent introduction of atezolizumab plus bevacizumab therapy resulted in further tumor shrinkage and hypovascularization. **c** The S7 nodule continued to grow throughout the treatment period; however, proton beam therapy (PBT) led to a reduction in size and hypovascularization of this lesion. AFP, alpha-fetoprotein; PIVKA-II, protein induced by vitamin K absence or antagonist-II

**Fig. 2:**
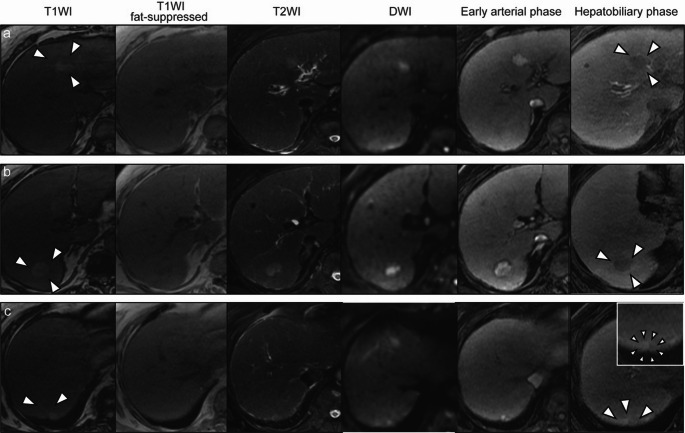
Magnetic resonance imaging (MRI) assessment of the S4, S6, and S7 nodules at the initial examination. Nodules in the **a** S4, **b** S6, and **c** S7 demonstrated the following characteristics: hyperintensity on T1-weighted imaging (T1WI), isointensity on fat-suppressed T1WI, hyperintensity on both T2-weighted imaging (T2WI) and diffusion-weighted imaging (DWI), early enhancement in the arterial phase. In the hepatobiliary phase, the S4 and S6 nodules demonstrated decreased uptake with low signal intensity, while the S7 nodule retained weakly enhanced signal intensity with less reduction in uptake. In panel (c), the top‑right inset provides the enlarged hepatobiliary phase view. White arrowheads indicate the nodules. Most of these imaging features were consistent with hepatocellular carcinoma (HCC)

The blood tests revealed modest hepatic dysfunction, corresponding to Child–Pugh class A. However, levels of alpha-fetoprotein (AFP) and protein induced by vitamin K absence or antagonist-II (PIVKA-II) were both normal. The blood test results obtained upon admission are presented in Table 1. The abdominal CT scan revealed the 25 mm nodule at S4 and the 19 mm nodule at S6. There was also a new 12 mm nodule at S7 (Fig. 1a-c). In addition, multiple viable small lesions were detected throughout the liver. On Gd-EOB MRI, these nodules were highly enhanced on T1-weighted imaging (T1WI), low-signal on fat-suppressed T1WI, high-signal on T2-weighted imaging, high-signal on diffusion weighted imaging, strongly enhanced in the arterial early phase. In the hepatobiliary phase, the S4 and S6 nodules showed low signal intensity consistent with decreased uptake, whereas the S7 nodule demonstrated weakly enhanced signal intensity with only minimal reduction of uptake (Fig. 2a-c). Neither extrahepatic lesions nor intrahepatic vascular invasion were detected on both CT and MRI.

**Table 1:** Laboratory serum test results

Peripheral blood		Biochemistry	
WBC	5600 /μL	Na	144 mmol/L
RBC	4.18 × 10^6^ /μL	K	3.5 mmol/L
Hb	13.7 g/dL	Ca	8.9 mg/dL
Plt	108 × 10^3^ /μL	Albumin	3.7 g/dL
Coagulation		Phosphorus	7.6 mg/dL
PT	15.4 s	UN	8.9 mg/dL
PT-INR	1.26	Creatinine	0.49 mg/dL
APTT	29.3 s	Total bilirubin	1.3 mg/dL
Fibrinogen	261 mg/dL	Direct bilirubin	0.3 mg/dL
FDP	1.9 mg/mL	AST	99 U/L
D-dimer	0.6 mg/mL	ALT	39 U/L
Viral markers		LD	231 U/L
HBs-Ag	< 0.05 IU/mL	ALP	321 U/L
HBs-Ab	< 10.00 mIU/mL	γ-GTP	141 U/L
HBc-Ab	7.72 C.O.I	CRP	0.22 mg/dL
HCV-Ab	(–)	Hyaluronic acid	129 ng/dL
Tumor makers		Type IV collagen 7S domain	9.1 ng/mL
AFP	7.9 ng/mL	M2BPGi	5.15
AFP-L3	5.5%		
PIVKA-II	14 mAU/mL		

Blood test results at the time of admission. WBC, white blood cell; RBC, red blood cell; Hb, hemoglobin; Plt, platelet; PT, prothrombin time; PT-INR, prothrombin time-international normalized ratio; APTT, activated partial thromboplastin time; FDP, fibrinogen/fibrin degradation products; HBs-Ag, hepatitis B surface antigen; HBs-Ab, hepatitis B surface antibody; HBc-Ab, hepatitis B core antibody; HCV-Ab, hepatitis C surface antibody; AFP, alpha-fetoprotein; PIVKA-II, protein induced by vitamin K absence or antagonists-II; UN, urea nitrogen; AST, aspartate aminotransferase; ALT, alanine aminotransferase; LD, lactate dehydrogenase; ALP, alkaline phosphatase; GTP, glutamyl transpeptidase; CRP, C-reactive protein; M2BPGi, mac-2 binding protein glycosylation isomer

**Fig. 3:**
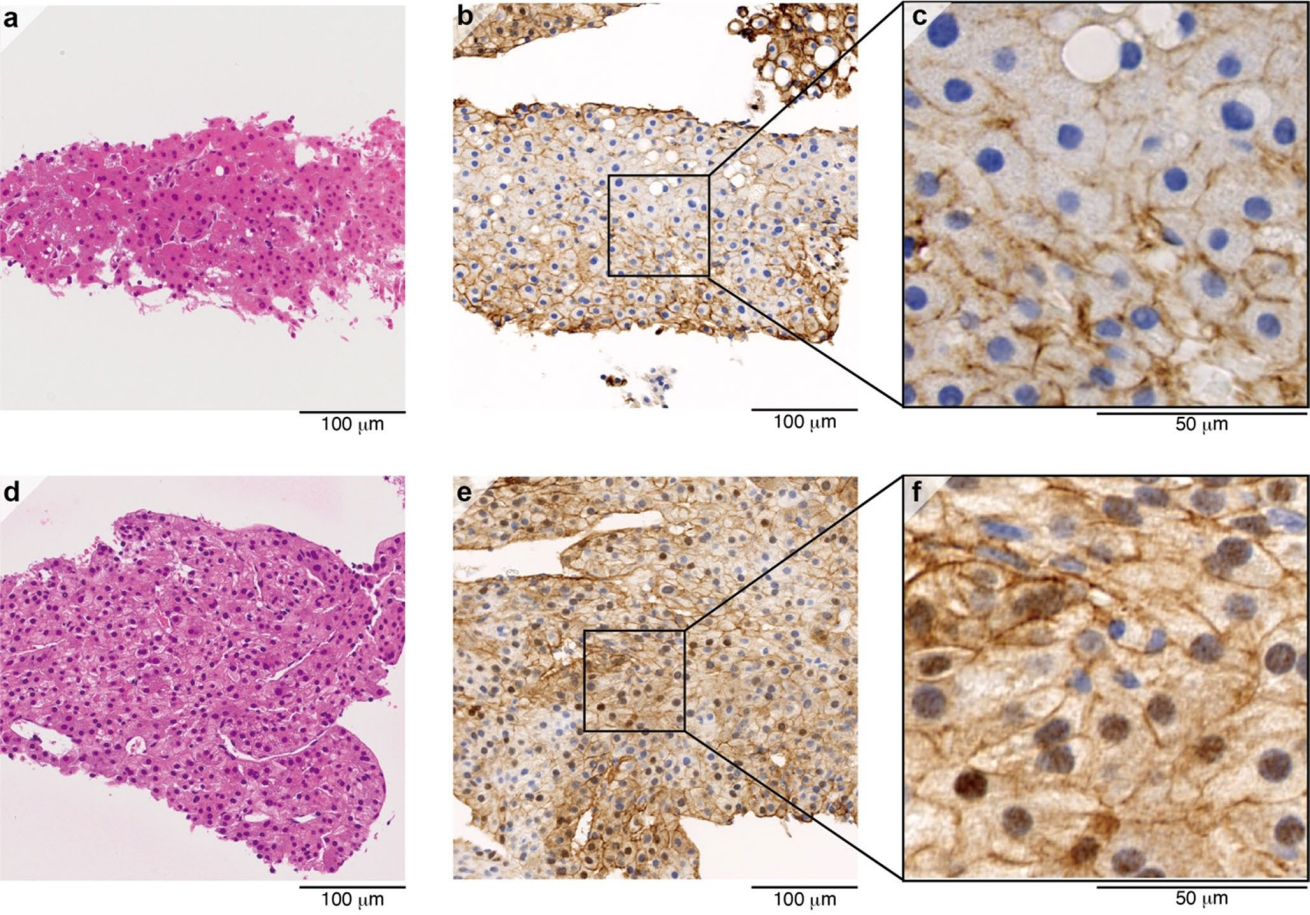
Immunohistochemical staining for β-catenin in liver nodule biopsies. **a**–**c** Biopsy samples obtained from the S6 nodule prior to treatment. The S6 nodule was negative for β-catenin. **d**–**f** Biopsy samples obtained from the progressively-enlarging S7 nodule at 31 months following treatment initiation. Most tumor cells demonstrated nuclear β-catenin positivity. **a** Hematoxylin and eosin (H and E) staining, × 20. **b** β-catenin staining, × 20. **c** β-catenin staining, × 80. **d** H and E staining, × 20. **e** β-catenin staining, × 20. **f** β-catenin staining, × 80

While the imaging results were mostly consistent with HCC, we proceeded with a percutaneous ultrasound-guided biopsy of the S6 nodule for histological confirmation, considering the absence of any elevation in characteristic tumor markers. Analysis of a biopsy specimen obtained from the S6 nodule showed a typical image of a well-differentiated HCC with proliferation of atypical cells, multiple layers of hepatocellular carcinoma, and structural disorganization (Fig. 3a). The background liver showed cirrhosis with extensive fibrosis and inflammatory cell infiltration in the portal vein area, while the hepatocytes had the appearance of alcoholic hepatitis with fatty deposits, overall classified as A2F4 according to the New Inuyama classification [12]. The S6 nodule was histologically confirmed as HCC. The S4, S7, and other micronodules were also diagnosed as HCC based on imaging findings, referring to histological features of the S6 biopsied lesion. Based on imaging findings of those HCC nodules such as affecting multiple segments in her liver and the absence of portal vein invasion, the patient was diagnosed with MO-HCC, developed on a background of alcoholic cirrhosis [13, 14].

As her hepatic function was reserved, she was classified to have BCLC stage B. Her condition met the up-to-7 criteria out; thus, a course of systemic chemotherapy was decided upon [4]. The treatment course and the changes in each particular nodule at S4, S6 and S7 over time are illustrated in Fig. 1. In December X, lenvatinib was initiated (atezolizumab plus bevacizumab was not indicated for HCC in Japan and was the first-line drug at the time). After initiating lenvatinib therapy, the AFP levels showed a progressive increase. A subsequent MRI scan in February X + 2 revealed disappearance of the micro-nodules, while the S4 and S6 nodules had become hypovascular. However, the size of the nodule at S7 had increased from 12 to 32 mm. Based on response evaluation criteria in solid tumors (RECIST) criteria [15], the patient was diagnosed with progressive disease (PD) and transitioned to atezolizumab plus bevacizumab therapy, which had passed adaptation for HCC in Japan [6]. After starting atezolizumab plus bevacizumab therapy, the AFP levels returned to normal. In total, 34 courses of atezolizumab plus bevacizumab therapy were administered; 22 of these courses involved atezolizumab alone, with bevacizumab omitted owing to the adverse effect of proteinuria. An MRI scan performed in April X + 3 revealed further diminution of nodules S4 and S6, but an increase in the size of the lesion in S7 from 32 to 40 mm. Due to the disparate response among those nodules to atezolizumab plus bevacizumab therapy, a liver tumor biopsy of S7 nodule was conducted. Pathologically, the S7 nodule was diagnosed as an intermediate to highly differentiated HCC (Fig. 3d). In order to investigate the difference in treatment response, β-catenin staining was performed. The S6 nodule exhibited a negative expression of β-catenin, whereas the S7 nodule demonstrated a positive nuclear expression (Fig. 3b, c, e, f).

As the other lesions were under control, PBT was administered to treat the lesion in S7 in June X + 3 following 20 courses of atezolizumab plus bevacizumab therapy. The combination therapy was subsequently restarted, and a CT scan in September X + 3 showed decreased contrast enhancement, which was suspected to be necrosis in the S7 nodule. Subsequently, a CT scan in June X + 4 showed tumor resolution. After completing an additional 14 courses of atezolizumab plus bevacizumab therapy (34 courses in total), the treatment was discontinued in July X + 4 based on the sustained normalization of tumor markers, complete disappearance of target lesions, and absence of any new HCC lesions. The patient currently remains in good general health with no recurrence.

## Discussion

The significance of ICIs in the treatment of unresectable HCC has increased concurrently with advancements in systemic chemotherapy for this condition. The combination therapy of atezolizumab plus bevacizumab has shown remarkable efficacy in improving both overall survival and progression-free survival, and has thus been established as a major treatment option for HCC [6, 7]. In our case, this combination therapy achieved good therapeutic outcome for multiple nodules. However, the S7 lesion alone showed treatment resistance; therefore, to uncover the molecular basis for the differential treatment responses, we performed a tumor biopsy of this lesion and compared its histological features with those of the pre-treatment biopsy samples from the S6 nodule, which had responded to atezolizumab plus bevacizumab therapy.

This biopsy evaluation proved crucial in determining subsequent treatment strategies. Immunohistochemistry further revealed that the S7 lesion was β-catenin positive, in contrast to the S6 lesion, which was β-catenin negative. Recent comprehensive analyses using next-generation sequencing have reported that activated mutations in the Wnt/β-catenin pathway was involved in ICI treatment resistance in HCC patients [16, 17]. Activation of β-catenin has been shown to correlate with CD8-positive T cell depletion and resistance to anti-PD-1 therapy [8, 17], which may explain the mechanism of poor treatment response in the S7 lesion in our case. Interestingly, these pathological findings were consistent with the pre-treatment Gd-EOB-DTPA-enhanced MRI results. The S4 and S6 nodules, which responded well to ICIs, showed low signal intensity in the hepatobiliary phase, whereas the ICI-resistant S7 nodule exhibited weakly enhanced signal. Previous studies have suggested that relative hyperintensity on the hepatobiliary phase predicts unfavorable response to ICIs [18, 19]. In addition, Wnt/β-catenin-activated nodules tend to show high signal intensity in the hepatobiliary phase of EOB-MRI [19, 20]. These findings may support the potential of EOB-MRI as a non-invasive biomarker for ICI resistance.

In multicentric HCC, it has been reported that individual nodules have different immunological backgrounds and show varying responses to ICI therapy [21, 22]. This may contribute to making treatment of multiple HCC complicated. In our case, the molecular biological and immunological heterogeneity of the tumors likely contributed to the differences in treatment effects. These findings support the proposal that β-catenin could serve as a predictive biomarker for ICI treatment response, especially in cases with multicentric HCC. In addition to the β-catenin/Wnt (CTNNB1) system, tumor-associated macrophages (TAMs), cytotoxic activity (CYT), and regulatory T cells (Tregs) may be involved in the immunological status of HCC, which could affect the efficacy of ICIs [23]. Although TAMs, cytotoxic activity, and Tregs were not directly evaluated in this case, their potential contribution to an immunosuppressive microenvironment cannot be ruled out. As MO-HCC may show heterogeneous immunological subclasses among nodules, integrated profiling of representative lesions may be warranted to guide personalized therapy.

Based on these findings, we selected PBT for the ICI-resistant S7 lesion. While PBT has been reported to show high local control efficacy and safety for HCC [24, 25], its combination with systemic therapy, including ICIs, has not been sufficiently investigated. In our case, we determined PBT to be appropriate based on three factors: (1) control of other tumor lesions, (2) manageable size for local therapy, and (3) the good general condition of the patient. Although other local options, such as radiofrequency ablation (RFA) and transarterial chemoembolization (TACE), were considered, we did not proceed with RFA because adequate local control was considered unlikely given the tumor size (approximately 40 mm) [6]. TACE was also evaluated; however, preprocedural hepatic angiography demonstrated reduced hepatoportal venous flow. As ischemic complications were a concern, TACE was deferred [26]. As a result, good local control was achieved for the S7 lesion. These results indicate that add-on PBT could be an effective treatment option for ICIs-resistant lesions in patients with MO-HCC. However, in Japan, insurance coverage for PBT is restricted to specific indications, which limits its widespread clinical use. Thus, while this case highlights the potential benefit of PBT, its application should be considered within the constraints of current healthcare policy.

Here, we describe a case of multicentric HCC with various intra-nodal expression of β-catenin. This case further demonstrates the importance of molecular biological and immunological heterogeneity in MO-HCC and the significance of personalized treatment based on these characteristics. However, as this is a single case report, the findings should be interpreted with caution. Further investigations involving larger cohorts are required to validate β-catenin as a routine predictive biomarker for ICI resistance.
